# QuPath: The global impact of an open source digital pathology system

**DOI:** 10.1016/j.csbj.2021.01.022

**Published:** 2021-01-21

**Authors:** M.P. Humphries, P. Maxwell, M. Salto-Tellez

**Affiliations:** a Precision Medicine Centre of Excellence, The Patrick G Johnston Centre for Cancer Research, Queen’s University, Belfast, UK; b Integrated Pathology Programme, Division of Molecular Pathology, The Institute of Cancer Research, London, UK

**Keywords:** QuPath, Digital Pathology, Computational Pathology, Image Analysis, Artificial Intelligence

## Abstract

QuPath, originally created at the Centre for Cancer Research & Cell Biology at Queen’s University Belfast as part of a research programme in digital pathology (DP) funded by Invest Northern Ireland and Cancer Research UK, is arguably the most wildly used image analysis software program in the world. On the back of the explosion of DP and a need to comprehensively visualise and analyse whole slides images (WSI), QuPath was developed to address the many needs associated with tissue based image analysis; these were several fold and, predominantly, translational in nature: from the requirement to visualise images containing billions of pixels from files several GBs in size, to the demand for high-throughput reproducible analysis, which the paradigm of routine visual pathological assessment continues to struggle to deliver. Resultantly, large-scale biomarker quantification must increasingly be augmented with DP. Here we highlight the impact of the open source Quantitative Pathology & Bioimage Analysis DP system since its inception, by discussing the scope of scientific research in which QuPath has been cited, as the system of choice for researchers.

## 1 Introduction

The use of open source software is becoming a key component of modern scientific activity. Indeed, there is increased evidence that some of the key discoveries in many areas of science would have not been possible without open source tools [1]. Of the thousands of scientific applications world-wide, the use of open practices and open resources in the field of digital pathology has revolutionizing tissue-based image analysis [2]. In areas such as cancer diagnostics and cancer research, there is an increasing interest in analyzing how these practices are dictating patient management and patient stratification [3]. We hereby analyze how QuPath, arguably the most widely used image analysis software in the world, has impacted the quantitative analysis of tissues and cells in research and diagnostics, as a way to illustrate how these tools are influencing the delivery of contemporary research.

QuPath, short for Quantitative Pathology, is an open source software with an active and engaged community able to support the development of tools for image analysis. The platform allows researchers with software development skills to add their own extensions to solve new challenges, but was designed for users without computer programing skills. QuPath is able to exchange data with existing tools such as ImageJ and MATLAB, and while ImageJ is perhaps the best known open source software for biomedical image analysis, it has historically struggled to deal with the size of whole slides images (WSI). On the back of the explosion of DP and a need to comprehensively visualise and analyse WSIs, QuPath was developed to address the many needs associated with tissue-based image analysis [4]. These needs were several fold and predominantly translational in nature: from the requirement to visualise images containing billions of pixels from files several GBs in size, to the demand for high-throughput reproducible analysis, which manual pathological assessment continues to struggle to deliver [5], [6], [7]. Resultantly, large-scale biomarker quantification must increasingly be augmented with DP.

QuPath was designed with the user in mind, with an easy-to-use interface that provides researchers and diagnosticians an ability to easily navigate complex tasks, such as automatically detecting many thousands of objects, classify and count these across large images, all while providing flexibility and high-throughput automated processing capabilities. Specific documentation on the introduction to analysis, installation, useful tutorials and much more is maintained and curated by the lead creator and developers of QuPath and can be found here: https://qupath.readthedocs.io/.

Due to its comprehensive nature as a tool for working with WSI, QuPath is a cross-platform software application designed for bioimage analysis that can be applied to numerous types of images beyond pathology. Although primarily created and used in cancer research for high throughput biomarker analysis in immunohistochemically stained formalin fixed paraffin embedded tissues, QuPath is able to meet the needs of many users: from laboratory researchers wishing to obtain raw quantitative data, to computational scientists working on the development and testing of algorithms. Primarily, QuPath software was designed for WSIs in digital pathology, to enable the analysis of immunohistochemistry (brightfield or fluorescent) and haematoxylin and eosin (H&E) images. QuPath’s interactive interface is user-friendly and is able to count cells, and classify objects and pixels in large WSIs without the need for cropping or down‐sampling images to lower file sizes for subsequent analysis.

Herein, we highlight the global impact of QuPath since its inception and discuss the scope of scientific research in which QuPath has been cited as the system of choice for researchers. Lastly, we aim to illustrate the need for reliable analysis in a digitised framework especially with regard to the development of artificial intelligence (AI) models.

### 1.1 The application of QuPath

Detailed information, including specific resources for support, documentation, compatible file types and instruction on the use of QuPath are available https://qupath.github.io/, https://qupath.readthedocs.io/, https://www.youtube.com/c/QuPath and are considered beyond the scope of this article. Broadly speaking, the software can support a wide variety of applications. QuPath is able to open and present WSIs from a wide range of file type’s from many digital pathology scanners. It is perhaps important to highlight that QuPath does support the Digital Imaging and Communications in Medicine (DICOM) standard format for WSIs. Users can view associated properties of an image, and using intuitive controls and gestures, navigate around images, panning and zooming as needed. Users can easily annotate tissues manually or with automated processes. Detection of cells is effortless with in-built steps and helpful default settings. Users can simply view measurements and export data. While performing analysis in this interactive step-wise method has its uses, the software contains the potential to create and apply similar analysis in a reproducible batch processing manner across a large image sets.

The flexible WSI viewer, which incorporates specific tracking slide navigation, is the cornerstone of many other relevant tools [8]. QuPath supports the quantification of hybridization signals with specific subcellular localization detail, automated tumour identification, tile-splitting of images to encourage faster analysis, estimation of stain intensity, capacity to exchange data with open source tools and the ability to read many image types, while carrying out real-time analysis with clinic-pathological parameters.

These automated processes are easily arranged into simple workflows created from the command history. As an example of a popular workflow within QuPath, the assessment of tissue microarrays (TMA) begin with the creation of an analysis project followed by automated de-arraying of the TMA and estimation of staining. A single‐cell detection is then undertaken, followed by trainable cell classification. Following biomarker detection, data may be further analysed within the software or exported. QuPath also facilities batch processing, and contains comprehensive survival analysis tools. Creation of workflows, allows the possibility to automate some or all of the analysis across multiple images. Viewing the command history allows for the creation of a script containing all the steps which have thus far been applied to an image. QuPath can be instructed to run a script over all project images to be analysed in a reproducible way. The flexibility of QuPath is continually evolving and scripting within QuPath can speed up analysis considerably. Advanced users recognise that scripting is able to unlock a huge range of features and possibilities.

### 1.2 The need for reliable analysis in a digitised framework.

The need for reliable analysis in a digitised framework is applicable to research and clinical applications, including diagnosis [9] and prognosis [10]. The increasing interest in digital pathology as a tool that supports discovery and delivers clinical utility may have arisen from the series of technical improvements which have transformed DP over the last 40 years into a reliable application [11]. These improvements and transformations include high-quality whole slide scanners, adequate image sizing and compression, speed and automation of the scanning process, and numerous options for image storage, and real-time retrieval/review. These technical developments have enabled DP to be the conduit to bring quantitation to a paradigm previously dominated by subjectivity. Quantitation such as: measuring nuclear morphology and DNA content; quantitative immunohistochemistry; analysis in multiple formats (e.g. tissue microarrays, appreciation of tumour heterogeneity and phenotypic variation); and capacity to identify and quantitate both chromogenic and fluorescent signals is swiftly achieved using DP. Resultantly, DP is able to overcome one of the main challenges we face in tissue analysis today, namely quantitative biomarker discovery. Indeed, DP can in parallel support the drug development and associated biomarker analysis process [11].

In the research environment, it is now clear that the need for more complex image analysis is growing exponentially and is transforming tissue-based discovery. These needs include an evaluation of broad topographic components of disease; for instance, the tumour stromal ratio in cancer samples or the characterization of tumour budding at the epithelial-mesenchymal transition [12]. A clear example of the need for image analysis and the application of deep learning to extract sub-visual features which can inform our understanding of geospatial variability of host immunity, is typified by the work of AbdulJabbar et al. [13]. In this study, the authors created a deep learning pipeline that enabled the spatial mapping of four distinct cell types within H&Es: malignant epithelial cells; lymphocytes; and non-inflammatory stromal cells, with an additional class of other non-identifiable cells or less abundant cells. This workflow collectively elucidated immune-evasive tumour microenvironmental mechanisms that may permit the emergence of aggressive clinical phenotypes [13]. The complexity and importance of the prognostication of spatial relationship is being further elucidated within the literature [14], [15]. Recently, Tsakiroglou et al. concluded that the proximity (<30 µm) of CD8 t-cells to PD-L1+ cells, as well as PD-1+ cells to PD-L1+ cells, was prognostic for overall survival in patients with head and neck squamous cell carcinoma. The authors cite QuPath’s custom scripting capability, active supportive community, well-maintained documentation, version management and open source nature as rational for utility with in their study.

Through the quantitation of tissue-hybridisation signals for specific biomarkers to confirm prognostic molecular pathways [10], and diagnostic solutions [16], we have shown how image analysis can greatly inform tissue-based discovery. Taking a robust digital pathology approach, we evaluated the suitability of alternative immune classifiers on prognostication in two independent colorectal cancer cohorts. We went on to assess the relationship with biology amenable to targeted therapy in a clinical trial cohort [10]. We have additionally shown the application of image analysis beyond discovery into a clinical application is not only feasible but much needed in the improvement of PD-L1 diagnostic accuracy [9], [16]. More generally, the application of image analysis and specifically deep learning, may be a prerequisite in the predication of molecular and outcome data from simple H&E images.

Sirinukunwattana et al. were able to take complex tissue organisation features from unclassifiable or heterogeneous cases of colorectal cancer and accurately predict RNA expression [17]. While not yet clinically viable, increasing the level of actionable data extracted from routine H&E slides, inaccessible to human interpretation, may providing vital information regarding tumour heterogeneity, with application potential in settings where previously there may have been no means of applying expensive molecular testing.

In the histopathological diagnostic setting, the need for digitisation and DP implementation cannot be overemphasised. Key elements to support such diagnostic decisions include [18]:

1. The need for accurate biomarker analysis in leading reference hospitals. Clinical trials in tertiary healthcare are in urgent need of digitisation, and in many cases are a *conditio sine qua non* for trial delivery
2. The need for reproducibility, consistency and accuracy in phenotypic diagnostics
3. The relevance of DP as a cost-effective tool, with formal accreditation by quality agencies
4. The need of a digitised service to apply AI solutions
5. The requirement for solutions allowing remote pathology diagnosis in the context of pandemics

It is in this fertile ground that we believe the numerous advantages of QuPath have flourished to support basic, translational and clinical research.

## 2 Methodology

The original paper [4], cited anywhere between 400 and 679 times at the time of writing according to a Web of Science Core Collection [19], and Google Scholar Cited Reference Search, spans many disciplines. Due to the prevalence of pre-print articles returned via Google Scholar, which are defined as preliminary reports that have not been peer-reviewed, we have chosen to focus on the conservative citation number from Web of Science, with the caveat that QuPath has been utilised in many more publications than cited. Our data does not include the frequent use of QuPath in abstracts for major scientific conferences such as USCAP and AACR meetings which we acknowledge are numerous.

### 2.1 The spread of QuPath

**Fig. 1:**
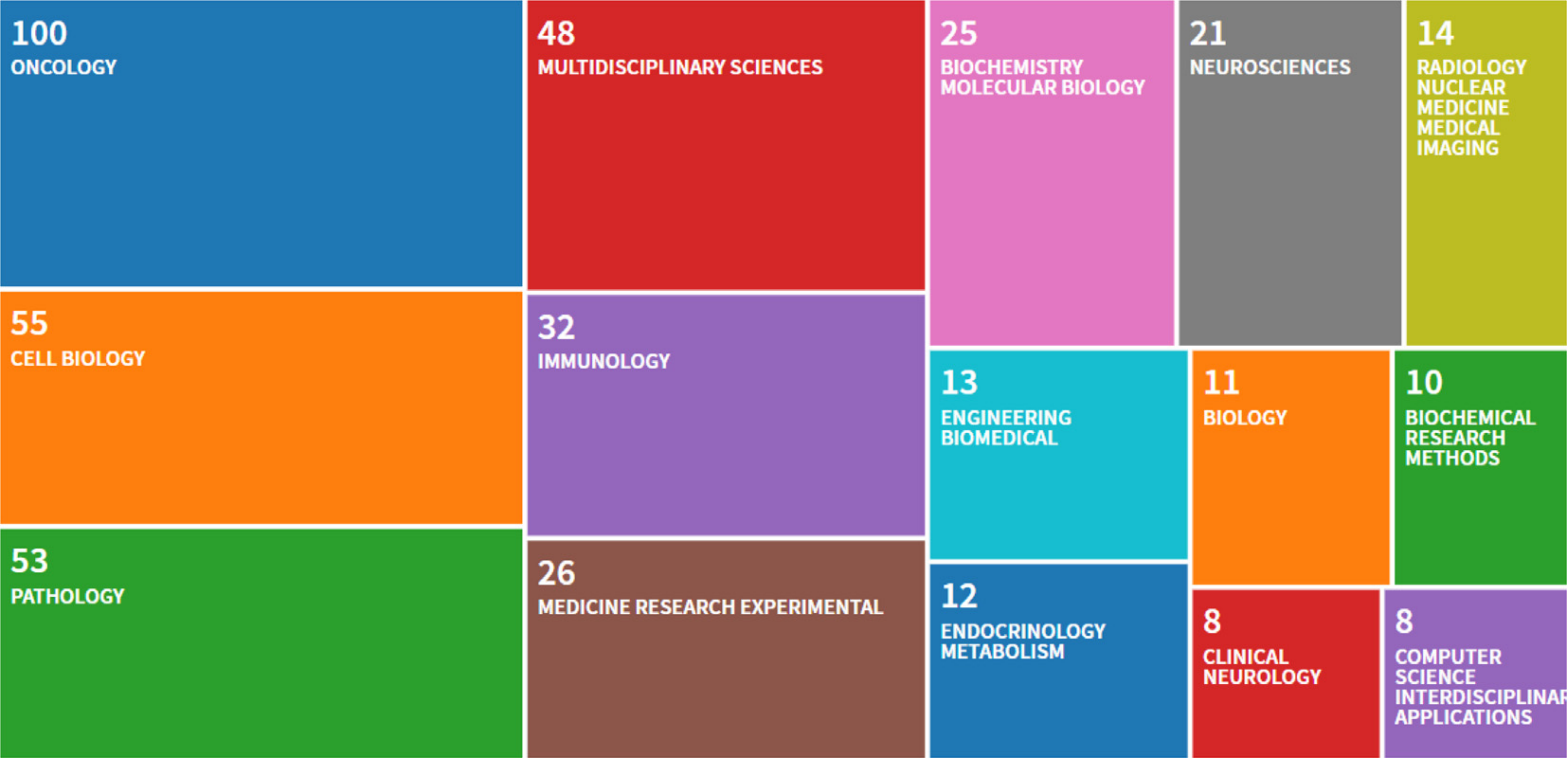
QuPath Research Areas. A treemap visualisation of the 15 top research areas assigned to the publications citing the seminal QuPath paper. Publications are not restricted to a single discipline.

The areas of specialization are numerous and span a broad range of disciplines from oncology to computer science, although it is perhaps unsurprising that oncology, cell biology and pathology are the more common areas of research that regularly utilise QuPath, as shown Fig. 1.

**Fig. 2:**
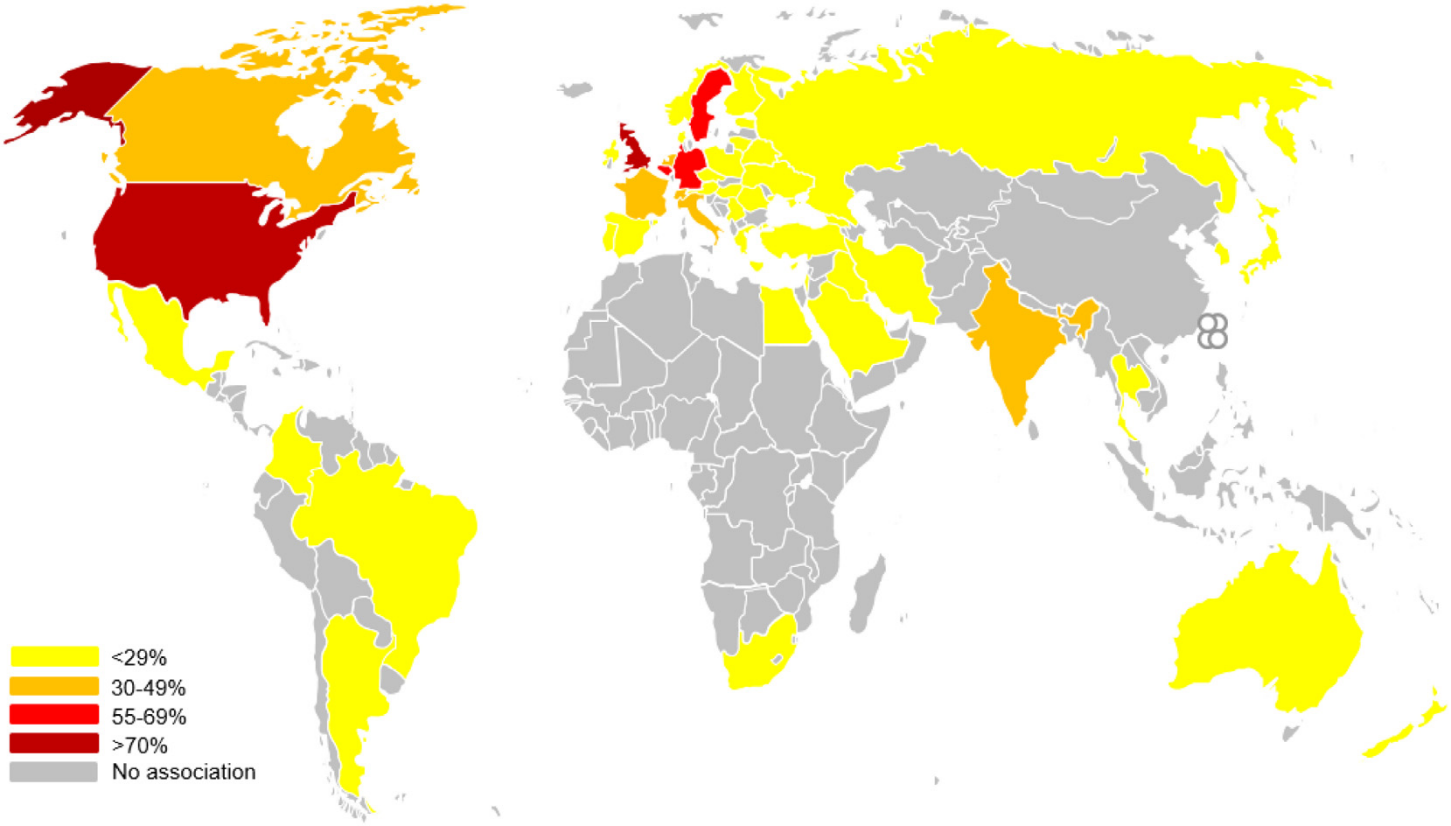
The Global Reach of QuPath. A visualisation of the regions and countries with institutional recognition of the use of QuPath, by way of publication.

Of these disciplines, there are >50 countries and regions represented that have published experience with QuPath in terms of the institutional affiliations associated with publications, (Fig. 2). Many of these articles associate with multiple countries for which the argument could be made that not only has DP facilitated and fostered national and international inter-laboratory collaborations, but owed to its open-source nature, that QuPath itself has connected institutions which otherwise would have little means of collaboration.

**Fig. 3:**
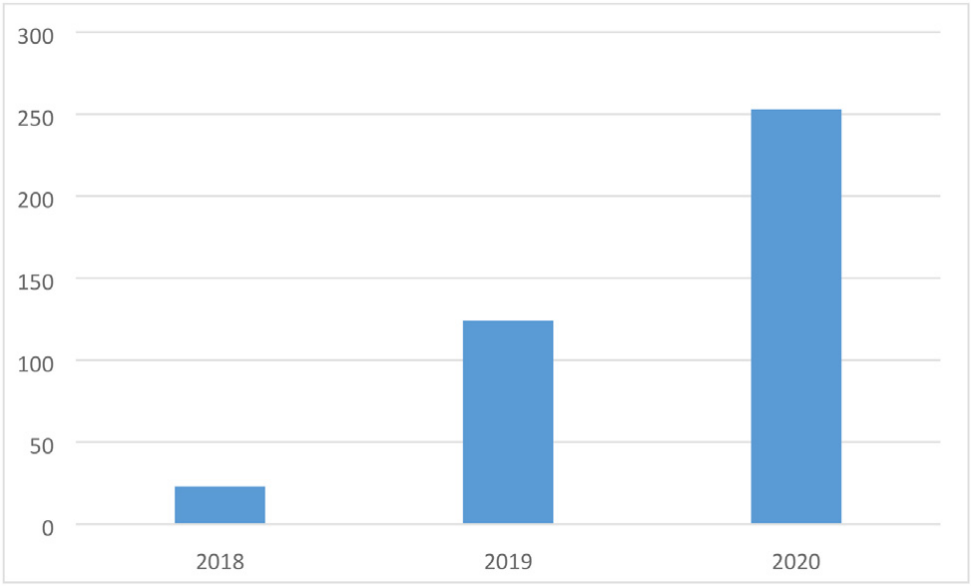
The Citation of QuPath Per Year. Web of Science Core Collection Cited Reference Search. Data last accessed 31/12/2020 11:00.

The utility of QuPath continues to grow unabated year on year, with publications growing at a rapid pace (Fig. 3). A cursory search of PubMed reveals that each year more authors and institutions are utilising QuPath for their research, although citation of the primary source is sometimes lacking, e.g. 2017–2020 [20], [21], [22], [23].

In addition to published research, communication and knowledge gathered internally and throughout our CRUK Accelerator network (https://www.qub.ac.uk/research-centres/PMC/cruk-centres-network-accelerator-award/) and beyond, indicate that QuPath is routinely used for the scoring of stained biomarkers in clinical trial material. Indeed, we are aware of contract research organisations, charities and biopharma are beginning to have an interest in utilising QuPath in their data analysis pipelines. The impact of QuPath is seen not only across domains as indicated above, but across translation boundaries within specialisms. The application of QuPath is regularly seen at the basic research level [24], [25], also within the translational research sphere [10], [26], [27], and recently in the clinical domain [16], [28]. Our own experience with contract research has indicated that in addition to off-the-shelf image analysis solutions, requests for biomarker analysis increasingly specify the use of QuPath for quantification https://www.qub.ac.uk/research-centres/PMC/Filestore/Filetoupload,972238,en.pdf. From a cost-analysis perspective, the ability to utilise an open-source DP platform with non-inferior performance to off-the-shelf DP solutions is appealing. In one such inter-platform and inter-operator analysis using the proliferation marker Ki67, a key comparative study concluded that QuPath was indistinguishable from others [29].

### 2.2 QuPath, a community

Owing to the open-source nature of QuPath, motivated and like-minded individuals can come together to contribute and share their experiences of QuPath. User forums provide a conduit to share workflow scripts, opinions and plans with other community members, general users and users of other complementary valuable open source projects.

The dialogue between users serves as a platform for development of novel algorithms, workflows and workarounds (https://forum.image.sc/tags/qupath), as well as the identification of software “bugs” or glitches (https://github.com/qupath/qupath/issues). At a time in which intellectual property and software development appear to be an indispensable component in developing a reliable research tool, QuPath’s co-operative model provides a fresh alternative. A high-quality academic open-source development, sustained and improved by a community of users represents a pathway with many advantages such as: accessibility, affordability and access to constant development of the original published platform. This ecosystem is at the heart of the ongoing evolution of QuPath.

### 2.3 The impact of QuPath

QuPath’s adoption by research groups delivering highly impactful research is undeniable. The ability to reliably and reproducibly quantify biomarkers has enticed researchers to use QuPath from round the world. As demonstrated by its appearance in some of the most impactful publications in journals such as Nature [30], [31], [32], Cell [33], [34], [35], [36] and Science [37], [38]. Roberti et al. used QuPath to quantify the cell density of DAB-positive cCasp3 cells; these data in part contributed to the demonstration that immunogenic ileal apoptosis contributed to the prognosis of chemotherapy-treated colon cancer [30]. The ability to use QuPath to robustly quantify Ki67 is well documented [29], [39]. In one Nature study the authors used QuPath to quantify the frequency of Ki67 positive cells in the whole crypt region of small intestine samples; these data contributed to the revelation of a regulated dynamic neuro-immune circuit where a trade-off between innate immune protection and efficient nutrient absorption was found. Indeed, this mechanism may be effective for enhancing resistance to pathogens and in the treatment of metabolic diseases [31]. A less often published capability of QuPath is the ability to robustly quantify BaseScope and RNAScope, two applications which allow for the *in situ* visualisation of biological functional units such as cell junctions, and RNA. Again in Nature, QuPath was used in the field of multiple sclerosis to quantify the number of BaseScope-positive signals in annotated regions. These data contributed to evidence that oligodendroglial heterogeneity in multiple sclerosis may be important for understanding disease progression and developing therapeutic approaches (32).

In developing the prognostic use of QuPath as a tool for annotation training for deep learning, Liu et al. [40] showed in 843 samples that their network could stratify patients with nasopharyngeal carcinoma into a high risk group with shorter than 5 year progression free survival (p < 0.0001).

It is clear that the majority of publications have used QuPath in brightfield histopathological assessment of biomarkers in FFPE sections, be these in resections [39], [41], biopsies [42], [43], cytology specimens [9], [44], TMAs [10], [45], [46], [47], [48], or embedded cell culture models [49], [50]. This is in addition to the multitude of immunofluorescence applications of QuPath [14], [16], [36], [51], [52]. Yet the intuitive annotation and stain quantification capabilities have enabled the application of QuPath in other, perhaps unexpected domains. For example, QuPath was used to perform semi-automated boundary detection of particles from X-ray fluorescence images from the mining domain, i.e. the processing of extracting precious mineral resources such as gold and silver [53]. Here the authors used pixel counting and boundary detection using specific colour values in QuPath to estimate the optical density of relatively high-arsenic regions in images which are critical to assessing potential short- and long-term exposure health risks to humans and surrounding environments.

### 2.4 The flexibility of QuPath

QuPath has undoubted utility in the quantification of ‘routine’ DAB biomarkers [9], [10], [54], [55], [56], [57], including RNAScope [28], [58], [59], [60], and the ability to handle complex quantification of multiple immunofluorescence biomarkers [16], [23], [51], [61]. As we have seen in the examples above, QuPath provides a framework for the training, provision and application of advanced AI, beyond that of the inbuilt machine learning methodologies. This framework could consist of anything from the training of more advanced deep learning neural networks by way of pathologist annotation or patch extraction, to ultimately the visualisation of the deep learning models trained on data acquired outside QuPath [62], [63], [64], [65], [66], [67].

**Fig. 4:**
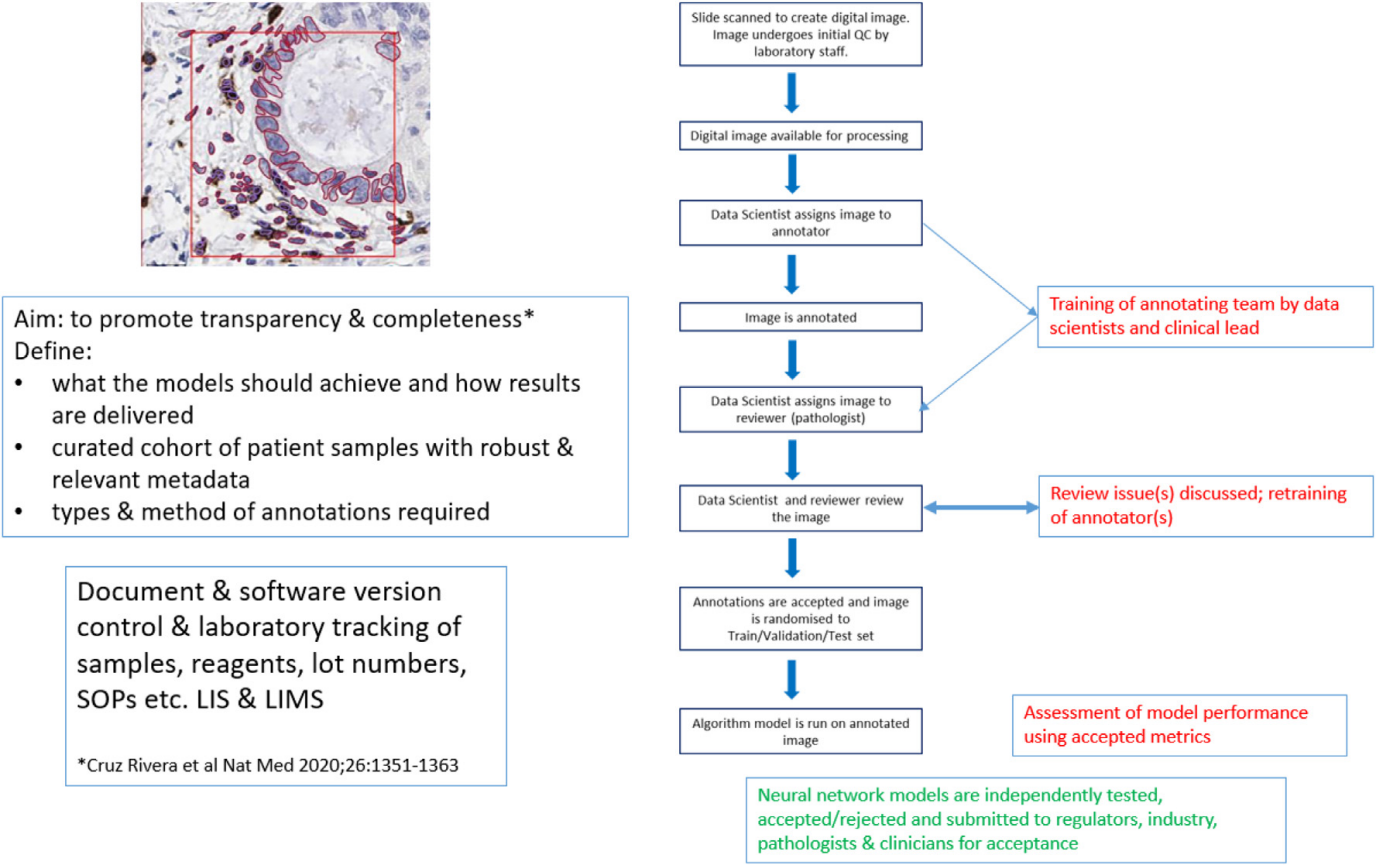
Translating research to practice. Workflow outline of using open software such as QuPath as a training tool for deep learning neural networks within a quality management system which maintains document and software control. Alongside training and competency of laboratory staff, annotators are trained and output is independently reviewed before being used in the training of the network. Change control is thus recorded to aid in the understanding of the development of the neural network for acceptance by regulators and the clinical community.

Using open source software for research purposes is useful, but translating such research to clinical utility poses challenges. A means of software version control is essential whereby changes in software versions are tracked, maintaining an understanding and traceability of how iterative versions of the same software differ one from another. In this way, regulatory bodies when reviewing software developments for use in clinical workflows can understand the mechanism by which a clinical decision is made, or trace any occurrences of clinical error, ensuring sufficient data for root cause analysis. Such measures required for the development of AI tools in clinical trials have been outlined [68]. The use of QuPath in the development of AI *via* deep learning techniques in a supervised or semi-supervised manner requires a level of version control to meet these challenges. The integration with a quality management system whereby documentation and version control is maintained, along with fully curated sample cohorts and robust metadata is matched with training and competency records of laboratory staff and those providing annotations. Independent and ongoing review of annotation data and feedback between data scientist and reviewer can identify any need for retraining of annotators (Fig. 4). In such a process version control of developing networks is possible. Tracking the version of QuPath used for annotations is recorded for the training and testing of neural networks, therefore resulting models can be version controlled, even when using open source software.

It is important to highlight here the need for widespread adoption of data version control (DVC) in the development and collaboration of AI models. DVC is a data versioning and experimental management tool, which builds upon established workflows. The sharing and collaboration often done through a standard Git-flow (i.e. commits, pull requests, etc.), can be combined with DVC to enable data scientists and machine learning teams to version control experiments, make projects reproducible and curate large datasets [69]. In addition to the accessibility of image training datasets and methods of classification, the quality of coding in AI and data driven machine learning applications is a pre-requirement for successful and sustainable software development. Property-based testing methodology ensuring code quality is necessary for the quality assurance, interpretation and tractability of graphical learning models [70]. These factors, as well as specifying programming languages utilised and clarifying whether these process are fully or semi-automated, need to be considered with a holistic appreciation for the complexity of a pathology department. While the specifics of any one element are vital decisions which must be taken for the introduction of AI and machine leaning, an end-to-end overview is necessary to appreciate the interdependencies of all workflow components [71].

As the third revolution in pathology evolves [72], the need for robust, and reliable bio-imaging tools will become necessary, and one where users of QuPath can rise to meet the challenge (Fig. 4).

### 2.5 Abstract and outlook

Disruptive technologies are often embraced by many, while simultaneously resisted by those whose faith in the prevailing paradigm is based on years of tried and tested methodologies. Since its inception, QuPath has achieved a remarkable uptake in its use across the domain of pathology. However, a limitation of our review of this space fails to capture the use of QuPath beyond that collated within publication repositories, such as the impact of QuPath within pathology education or the specific number of users within the biopharma industry. More recently, the flexibility of the system has appealed to researchers from other disciplines. Within a discovery research setting, it is clear that image analysis, and the application of deep learning has clear utility and robustness. The challenges facing clinical pathology is whether a seamless integration of image analysis into a digitised service is not only reproducible, consistent and accurate, but that it can be user-friendly, cost-effective and fold seamlessly into the framework of accreditation laboratories, while adding substantive value in supporting clinical services. Furthermore, improvements in explainable and transparent AI away from the perception of a black-box is essential for such models to be not only usable but also useful to the expert. The usability of AI models will build trust overtime allowing measurement of effectiveness, efficiency and satisfaction for users [73]. Whether QuPath may play a role in this future remains to be seen.

The concept of using open-source software either as stand-alone machine learning quantitative tools, or as a means to support the development of AI for clinical deployment will need to be used within a version controlled quality management system. An environment which can be validated and critically evaluated by regulators, pharma and co-industrial partners, and the clinical community. Commercial platforms are therefore seen as having the support from commercial-grade & quality assured frameworks and are perhaps better positioned to support image analysis for clinical utility. What is in no doubt, is that the development and application of image analysis and the development of AI will continue unabated. The translation of these tools from a research setting into a clinical setting through acceptance by regulators, industry and clinical groups is key to driving the next revolution in tissue based biomarker discovery.

The latest release of QuPath is accessible here: https://qupath.github.io/

## Funding

Funding information is not applicable.

## CRediT authorship contribution statement

**M.P. Humphries:** Conceptualization, Data curation, Formal analysis, Investigation, Methodology, Software, Writing - original draft, Writing - review & editing. **P. Maxwell:** Conceptualization, Investigation, Writing - review & editing. **M. Salto-Tellez:** Conceptualization, Funding acquisition, Investigation, Methodology, Project administration, Resources, Supervision, Writing - review & editing.

## Declaration of Competing Interest

The authors declare that they have no known competing financial interests or personal relationships that could have appeared to influence the work reported in this paper.
